# Do research collaborations age like wine? Absolute and relative measures of CANZUK research partnerships’ strength since the 1950s

**DOI:** 10.1371/journal.pone.0299319

**Published:** 2024-04-16

**Authors:** Ba Xuan Nguyen, Markus Luczak-Roesch, Jesse David Dinneen

**Affiliations:** 1 School of Information Management, Victoria University of Wellington, Wellington, New Zealand; 2 Faculty of Business Administration, Posts and Telecommunications Institute of Technology, Ho Chi Minh City, Vietnam; 3 Te Pūnaha Matatini, Aotearoa New Zealand’s Centre of Research Excellence for Complex Systems, Auckland, New Zealand; 4 School of Library and Information Science, Humboldt-Universität zu Berlin, Berlin, Germany; Instituto Tecnologico Autonomo de Mexico, MEXICO

## Abstract

Although previous studies of today’s globalised and competitive research landscape have mentioned the research collaborations of CANZUK countries (i.e., Australia, Canada, New Zealand, and the United Kingdom), none have yet studied them in detail. Further, such studies have used different measures of international research collaboration (IRC), resulting in disparate findings. This paper, therefore, analyses the strengths of CANZUK research collaborations, how those collaborations have changed over time, and assesses the effect of three ways of measures on the results (absolute strength, bilateral similarity, and multilateral similarity). We provide a detailed characterisation of the CANZUK research network and its relationships with partner countries, which reveals that the most collaborative CANZUK countries are the UK and Australia, among other findings. We also confirm that many findings differ depending on which measures are used. We offer an explanation of this difference with reference to the nature of the measures (i.e., what they really measure) and make suggestions for suitable measures in future studies depending on their purpose. Finally, we discuss how this study’s findings can be used by research policy makers (in CANZUK and elsewhere) in deciding on research strategy and by researchers in appropriately measuring IRC.

## Introduction

International research collaboration (IRC) has become a more developed and important topic in bibliographic research [1, 2]. Determining the patterns of research collaborations between countries is one core area of IRC inquiry [2]. This research is important for understanding the global scholarly landscape, with the potential to shed light on how international actually is and whether there are any imbalances and even inequities among scholars’ involvement, or their ability to be involved, in high-impact research partnerships based on their location.

In this paper we investigate IRC among the so-called CANZUK countries, Canada (CAN), Australia (AUS), New Zealand (NZL), and the United Kingdom (GBR). The countries in this study are referred to by their corresponding three-letter country abbreviations (in ISO-3166 Alpha-3 standard). The explanation of these three-letter country abbreviations is presented in S1 Table.

There are a number of discussions on how tight the pairwise connections between CANZUK countries have been [3, 4], and increasing interest in whether these connections will be tighter or looser after “Brexit” [5]. However, there has been no research studying the research collaboration (RC) of the countries in this cluster as a whole. Nor have there been studies on how these countries, which typically engage in high levels of research activity, are connected to other countries outside of the CANZUK cluster. Thus, an examination of the different ways of measuring CANZUK’s RC strengths is necessary because each way of measuring serves a different purpose [6] and results in a different IRC network map [7].

This paper aims to analyse the strengths of CANZUK research collaborations, and how these collaborations have changed over time. The paper is structured as follows: The ‘Related work’ section introduces a brief description of the fundamentals and related work of the measurement for IRC patterns. The ‘Materials and methods’ section reports the data and methods used in the study. The ‘Results’ section describes the main findings. The ‘Discussion’ section presents the discussions of this paper. The paper concludes by providing insights and recommendations for future research in the ‘Conclusion and future work’ section.

## Related work

### IRC networks and IRC patterns

Various properties of global collaboration networks have been uncovered in previous studies. Firstly, IRCs have been developing in a preferential attachment process, in which those countries that have already been research cores are continuing to develop more collaborations [8]. Secondly, these networks have been recognised as ‘‘small worlds” in which any two researchers are often connected by a short path of collaborations [9].

However, these networks have been described differently over different periods. In the period 1980–2000, the centre ‐ periphery relationship was examined for the IRC network [7]. Once the IRC research network had expanded with the participation of more nations, this global network also became more interconnected between 1990 and 2000 [8]. Some of the regional networks were considered semi-peripheral regions [10], and a more competitive multi-centric core was observed [11].

The comparison of countries’ scientific profiles has also been used to classify countries having similar IRC patterns side by side. A study by Okubo et al., which examined the publications of eight large scientific fields in the period 1981–1986, revealed a proximity map in which there were many clusters of countries’ scientific profiles [12]. One notable cluster includes Great Britain, which is the country at the centre of this proximity map, and the USA, Canada, Australia, New Zealand and Nigeria. Although the proximity map suggests possible historical effects shared by the countries having similar patterns of countries’ scientific profiles, it does not mean these countries have close research relationships.

### Mapping IRC networks and the CANZUK research cluster

Graphical presentations of IRC networks or network ‘maps’ are useful to provide an overview of IRC relationships. Co-authorship maps have been commonly used to present these IRC relationships. At the national level, each node in a co-authorship map represents a collaborative country and each connection represents the RC between a pair of countries. Four co-authorship clusters have been described with a tiny one including Australia and New Zealand, and this tiny cluster connects to the Western European cluster through the Australia-UK connection [13–15]. A co-authorship map describes IRC’s scientific factors through the sizes of its nodes (describing the total numbers of countries’ IRCs) and its connections (describing the numbers of IRCs between collaboration pairs).

Although a separate research cluster including Australia and New Zealand has been mentioned in prior studies, little is known about the RCs among CANZUK countries, and the RCs between the CANZUK members and other countries. Therefore, it is unclear for example, whether the conclusion that the IRC of GBR has been impacted more by historical connections than geographical proximity [16] may be true just for GBR or for the other CANZUK members as well.

### IRC measures

IRC studies have used different measurements to analyse the research collaboration patterns across countries. Table 1 shows the representative IRC pattern studies listed by Chen, Zhang, and Fu [2], and the corresponding measurements used in these studies.

**Table 1:** Different measurements have been used in representative IRC pattern studies.

Representative IRC pattern studies (title)	Measurements used
“International collaboration in the sciences 1981–1985” [13]	Salton’s measure
“International collaboration of three East European countries with Germany in the sciences, 1980–1989” [17]	Salton’s measure
“Transnational linkages of Indian science: A structural analysis” [18]	Internationalization Index (INI), Cooperation Index (COI), Cooperation Extensiveness Index (CEI) and Affinity Index (AFI)
“A study of collaboration in laser science and technology” [19]	International Collaboration Index
“Mapping world scientific collaboration: Authors, institutions, and countries” [20]	Number of international publications
“Impact analysis of domestic and international research collaborations: a Malaysian case study” [21]	Internationality index
“A bibliometric analysis of lab-on-a-chip research from 2001 to 2013” [22]	Total number of (international collaborative) publications

There are generally three different approaches to quantify the RCs between countries [7, 13, 14, 23–26]. The first approach is the ‘absolute strength’ measure [13, 27], implemented by counting the bilateral RCs’ observed frequencies. The meaning of the term ‘strength’ used in this context is ‘activity’ of research collaboration [7]. The other approaches are in the group of ‘relative strength’ measurements. To measure the relative strength of scientific linkages in science, the measurements are either size dependent (e.g., AFI which takes the total co-authorship linkage of one country into calculation) or size-independent (e.g., Salton’s measure which normalises the size of total co-authorship linkage of both countries under survey). While the size dependent measurements emphasise on the important research partners, the size-independent measurements demonstrate the links [28] between countries’ IRC strength and their proximities (e.g., cultural proximity or linguistic proximity). In this study, we aimed to explore the relative strength of countries in their global context, which led us to choose size-independent measurements. Within this group of size-independent measurements, two different approaches emerged: bilateral similarity measures, and multilateral similarity measures as follows:

The second approach is bilateral similarity measures [7, 8, 13, 29, 30]. In this approach, the observed bilateral RC numbers are adjusted by the total RC numbers of the two countries involved. There are many ways to implement this normalisation but the most popular ones in the field of scientometrics include the following four measures [31]:

The association strength:

$$A_{xy}=\frac{C_{xy}}{C_{x}C_{y}}$$

Inclusion index:

$$I_{xy}=\frac{C_{xy}}{\min\;\left(C_{x},C_{y}\right)}$$

Jaccard index:

$$J_{xy}=\frac{C_{xy}}{\min\;\left(C_{x}+C_{y}-C_{xy}\right)}$$

and Salton (cosine) index:

$$S_{xy}=\frac{C_{xy}}{{\left(C_{x}\;C_{y}\right)}^{1/2}}$$

where:

A_xy_: the association strength between two countries, x and y

I_xy_: the inclusion index between two countries, x and y

J_xy_: the Jaccard index between two countries, x and y

S_xy_: the Salton index between two countries, x and y

C_xy_: the observed number of their co-authored papers

C_x_ and C_y_ is the total number of IRC papers published by countries x and y,

respectively

Theoretically, the association strength measure is classified as a probabilistic similarity measure and the three remaining measures are classified as set-theoretic similarity measures. Probabilistic similarity measures "can be interpreted as measures of the deviation of observed co-occurrence frequencies from expected co-occurrence frequencies under an independence assumption", while set-theoretic similarity measures "can be interpreted as the relative overlap of two sets" [31]. Although probabilistic similarity measures seem to be more appropriate for normalisation purposes, the Jaccard index and the Salton cosine have been the two most popular measures in scientometric research [31].

The third approach is multilateral similarity measures. This approach relates the relative strength of two countries’ bilateral RCs to their collaborative relations with other countries [7, 8, 16, 28]. These relative strengths are calculated by getting the ratio of observed and expected values of bilateral RCs, in which the expected values are obtained with the following formula:

$$E_{xy}=\frac{C_{x}\;C_{y}}{T}$$

where:

E_xy_: the expected values of bilateral RCs between two countries, x and y, in comparison to collaborative relations with other countries.

C_x_ and C_y_ is the total number of IRC papers published by countries x and y, respectively

T: the total number of IRC papers of all countries.

Consequently, the collaborative networks produced by applying the above three measure approaches represent the symmetric relationships between countries. The reasons are because bilateral RCs between a pair of countries are counted twice, both as an incoming and outgoing edge in collaborative networks (in the ‘absolute strength’ measure), and the denominators in the formulas of these three measures use both the total numbers of IRC papers published by two countries as a way of normalisation (in bilateral similarity measures and multilateral similarity measures).

Two underlying counting methods have been commonly used in the above measures: the whole counting method and the fractional counting method [32]. Of these, the former measures the collaborative activities by assigning a full credit to each unique country having participants in a co-authored publication [12]. The latter measures the collaborative activities by assigning fractional credit to each country based on the proportion of authors it contributes to a given publication [7]. However, the number of collaborative activities alone cannot indicate how strong the RCs of the countries involved are, as measured by the group of ‘relative strength’ measurements. The reason for this is that a number of bilateral RCs considered large (i.e., to small countries) may be just a small RC number to big countries. In order to estimate the ‘propensities’ of RCs, for example, we need to examine the observed/expected ratio, so the countries’ sizes of IRC publications need to be taken into account.

The literature of IRC networks shows that there are research gaps about the CANZUK countries’ RCs, and how different measures can and should be used to reflect these RCs.

## Materials and methods

The purpose of the current study is to examine the CANZUK countries’ RCs by answering three RQs:

- *RQ1*: *How have the RCs among CANZUK countries developed over time*?
- *RQ2*: *How have the RCs between CANZUK and other countries developed over time*?
- *RQ3*: *To what extent do the different measures of RC reveal different IRCs*?

In this study, we used bibliographic data to quantify the RCs between countries. We also applied different ways (absolute strength, bilateral and multilateral similarities) to measure the RC strengths and to compare the results. The details are as follows:

### Data

**Fig 1:**
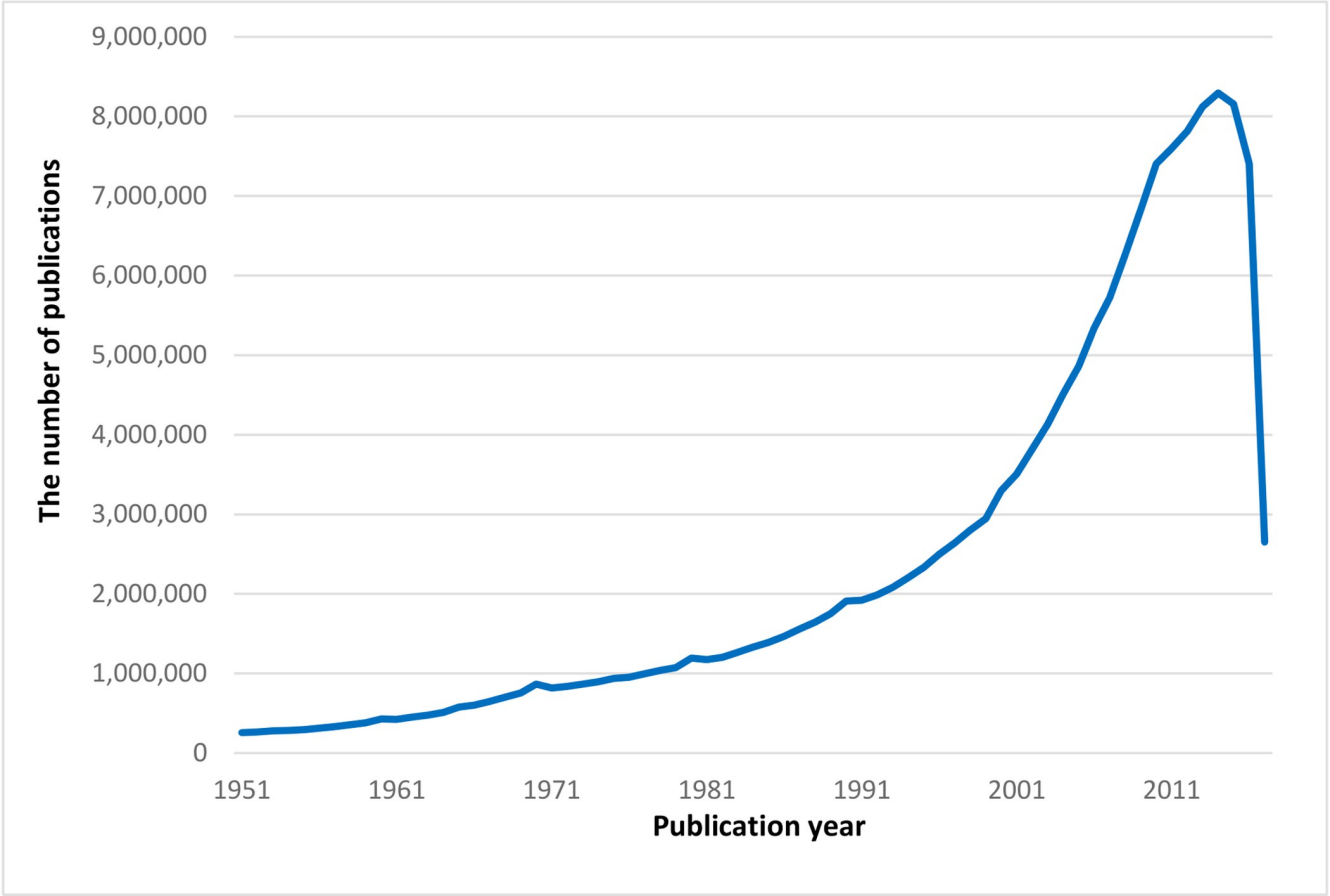
Annual production of scientific publications stored in the MAG data set during the time 1951–2017.

Due to budget constraints, we used Microsoft Academic Graph (MAG), a general scholarly bibliographic data set, to examine the historical research collaborations of CANZUK countries. The MAG data set is publicly available to researchers on the Microsoft website. For this study, the entire data set was extracted via bulk download. The data set encompasses publications from the year 1800 to 2018. Since not all publications from the year 2018 are entirely stored in this version of the MAG data set, the year 2018 was excluded from the study. A previous study [33] demonstrated that the mean number of coauthors in publications has only approached around two authors since the 1950s. Consequently, the period 1951–2017 was selected as the range of data for this study. The Fig 1 below shows the time analysis of the number of publications in the MAG data set in this period.

Given that most of IRC network studies have examined the collaborative network maps in the period 1981–2000 [2], we also aimed to compare the CANZUK’s RCs in this period with those in the previous and later periods. Therefore, the data from the years 1951 to 2017 were collected and separated into three periods: 1951–1980, 1981–2000, and 2001–2017. The Table 2 below displays the number and the percentage of publications in this data set, categorised by document type.

**Table 2:** Summary of different document types in the dataset of IRC publications used in this study.

Type of publications	Number of publications	Percentage of publications
‘Journal’	1,129,591	83.55%
‘Conference’	77,888	5.76%
‘BookReferenceEntry’	699	0.05%
‘Book’	503	0.04%
Unknown (null)	143,350	10.60%
***Total***	***1*,*352*,*031***	***100*.*00%***

The statistical software environment R with its *igraph* package was used to identify the RCs between countries from bibliographic information of co-authored publications in the data. We applied a method of completing authors’ country affiliation data in MAG records [34] to resolve missing data. Information about scientific factors ‐ numbers of total publications and international co-authored publications ‐ are extracted from this data source to investigate the research collaborations of CANZUK countries.

## Methods

We measured IRC in three different ways: absolute strength, bilateral similarity, and multilateral similarity to examine the development of research collaborations relating to CANZUK countries over time.

To measure the absolute strengths of RCs, we calculated the observed frequencies of RCs. We also analysed the distribution statistics of these RCs to estimate their relation patterns during these periods. To further compare the RCs of CANZUK’s countries, we measured the relative strengths of RCs using the following two ways:

To measure the bilateral similarity values of CANZUK’s RCs, we applied the most popular measures in scientometric research: the association strength, the inclusion index, the Jaccard index, and the Salton index. In the latter two measures, bilateral RCs having higher proportions reflect relatively more active connections between the two corresponding countries.

To measure the multilateral similarity of CANZUK’s RCs, the ratios of observed frequencies to expected frequencies of CANZUK’s RCs were calculated. Among them, those values greater than one imply that the corresponding RCs were more active than expected.

The underlying count used in the above measures could be either the whole counting or the fractional counting. The difference between these methods is that the former reflects the research connections between countries while the latter reflects the research productions (i.e., publications in this case) of the collaborative countries. For example, an article co-authored by one researcher from the UK and two researchers from the US will result in different counts. The whole counting method credits one for each country: the UK and the US. Meanwhile, the fractional counting method credits one half for the UK and one half for the US. Previous bibliometric studies made diverse arguments for using one or both of these counting methods [6]. Because a paper measuring the importance of CANZUK countries’ partners [32] showed that minor differences in results were obtained by applying the two different counting methods, we also applied both these two underlying counting methods to examine whether they affected the visualisations of collaborative networks, and compared the two sets of results.

The co-authorship map technique was applied in all these three different ways to visualise the collaborative networks among CANZUK countries, and the collaborative networks between them and other countries. With absolute strength and bilateral similarity, we converted the strength values to the ratios (of the strength values to the median values) to express the lines’ thicknesses. With multilateral similarity, the values were already expressed in ratios (of observed values to expected values) so conversion was unnecessary.

## Results

The results were presented in three sub-sections, which reflects the use of measures to evaluate the absolute strengths, bilateral strengths, and multilateral strengths of CANZUK’s RCs, as follows:

### Absolute strengths of CANZUK’s RCs

**Fig 2:**
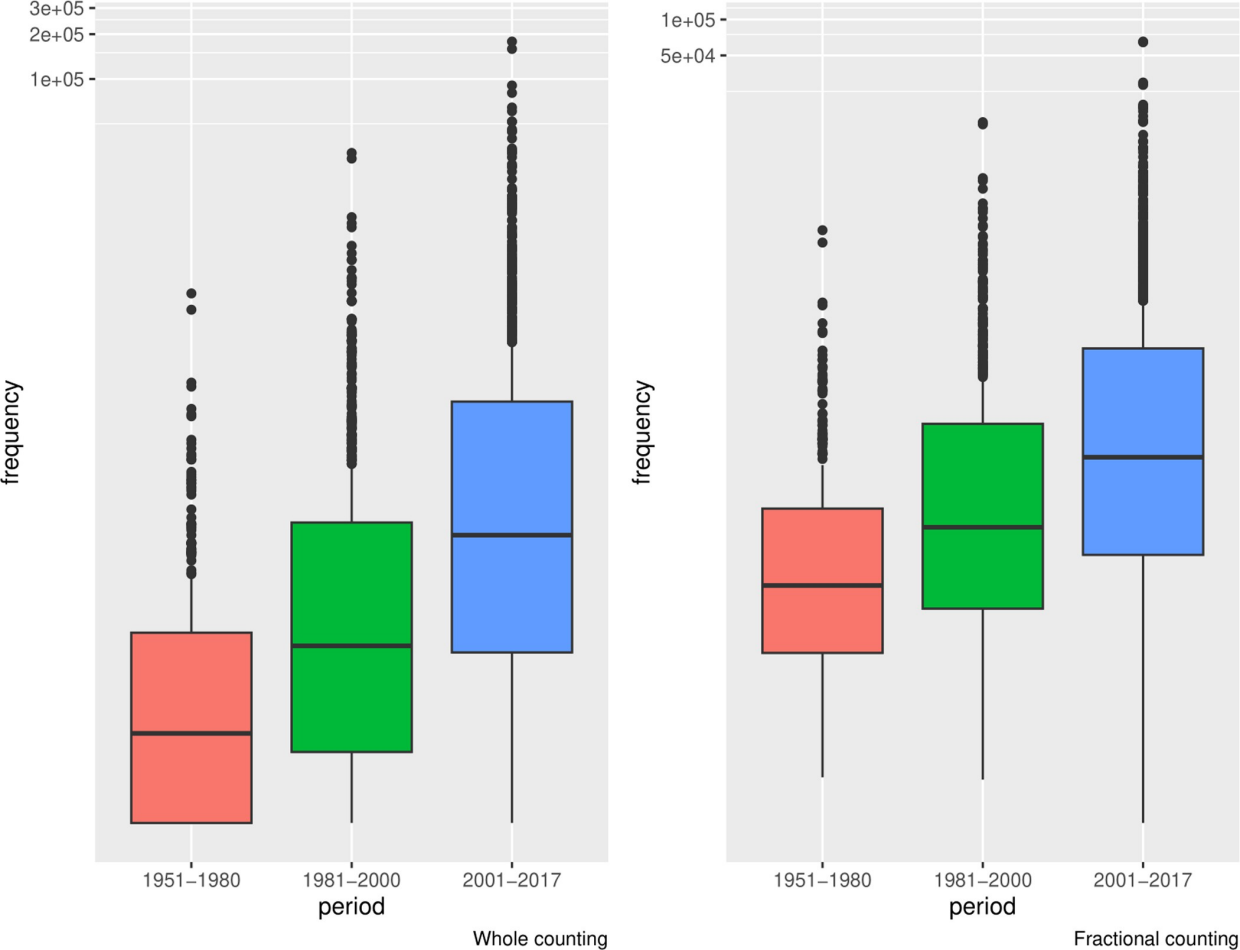
The distributions of co-authored publications involving CANZUK countries, calculated by the whole counting method and fractional counting method (The y axis is in log2 scale). The numbers of co-authored publications have increased during the period 1951–2017.

The observed frequencies of CANZUK’s RCs in the three periods show the following main points:

Firstly, CANZUK countries have increased research collaboration relationships over time (with each other or with non-CANZUK countries). This is presented by the increase of collaboration pairs (involving at least one country in the CANZUK group) during the period 1951–2017 (Table 3). The numbers of co-authored publications have also increased, represented by the median values of credits for co-authored publications in the same table. The increase of co-authored publications can be observed through the boxplots in Fig 2, which show that their interquartile ranges have generally moved up over time.

**Table 3:** The summary of research collaborations involving CANZUK countries in three separate periods during the time 1951–2017. The numbers of collaboration pairs and credits for co-authored publications between collaboration pairs, calculated by both whole and fractional counting methods, have increased over time.

Summary		Whole counting			Fractional counting		
		1951–1980	1981–2000	2001–2017	1951–1980	1981–2000	2001–2017
Number of collaboration pairs		363	658	893	363	658	893
Number of credits for co-authored publications between collaboration pairs	Max	3,622	31,851	178,569	1,724.54	13,831.21	65,428.91
	3rd quartile	19.00	104.50	679.00	8.07	41.33	176.73
	Mean	51.34	389.70	2,413.00	23.61	158.79	776.96
	Median	4.00	15.50	86.00	1.83	5.63	21.72
	1st quartile	1.00	3.00	14.00	0.50	1.17	3.31
	Min	1.00	1.00	1.00	0.05	0.04	0.02

**Fig 3:**
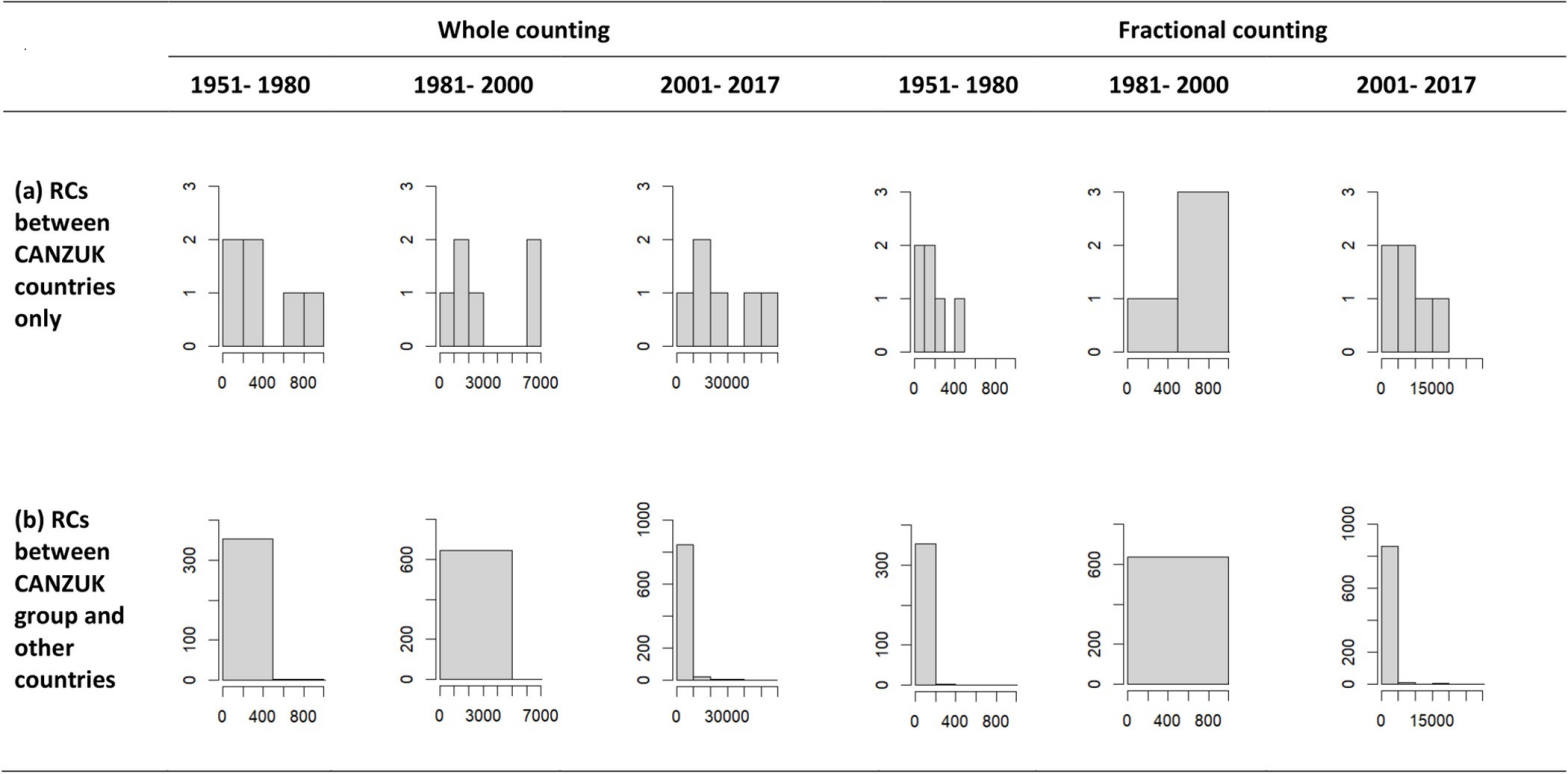
Histograms of distributions in the three periods of (a) RCs among CANZUK countries, and (b) RCs between CANZUK members and other countries (excluding the CANZUK only pairs). The distributions of (a) have a lighter tail while the distributions of (b) have a heavy tail.

Secondly, the distributions of RC strengths of CANZUK countries only, comprising six pairings between the four CANZUK countries, typically exhibit a shape with a low and wide peak, and a correspondingly thin tail on the right, as shown in Fig 3. Table 4 gives statistics of these distribution’s shapes. For the RC network of CANZUK countries only, the kurtosis values show that these distributions have a lighter tail than a normal distribution (kurtosis is less than 3). Meanwhile, the skewness values have decreased over time, indicating that these distributions have become more symmetrical.

**Table 4:** The distribution statistics of research collaborations of CANZUK countries in three separate periods during the time 1951–2017. The values show that the distributions of RC strengths among CANZUK countries and the distributions of RC strengths between the CANZUK members and other countries are different.

Metric	RCs in CANZUK countries only			RCs between CANZUK members and other countries (excluding the CANZUK only pairs)		
	1951–1980	1981–2000	2001–2017	1951–1980	1981–2000	2001–2017
Skewness (whole counting)	0.87	0.54	0.45	11.76	12.32	11.06
Skewness (fractional counting)	0.86	0.54	0.54	11.89	13.10	12.41
Kurtosis (whole counting)	2.23	1.52	1.60	152.69	180.83	156.59
Kurtosis (fractional counting)	2.22	1.56	1.90	154.79	199.89	191.08

Thirdly, the distributions of RC strengths between the CANZUK members and other countries, as illustrated in Fig 3 and detailed in Table 4, exhibit a sharp peak and a correspondingly fat tail. A difference is, unlike the distributions of RC relationships of CANZUK countries only having a lighter tail, the RC relationships between the CANZUK members and other countries have a heavier tail than a normal distribution (kurtosis is greater than 3). The heavy tails of distributions of RC relationships between the CANZUK members and other countries, as presented in Fig 3, mean that these distributions are outlier-prone.

**Fig 4:**
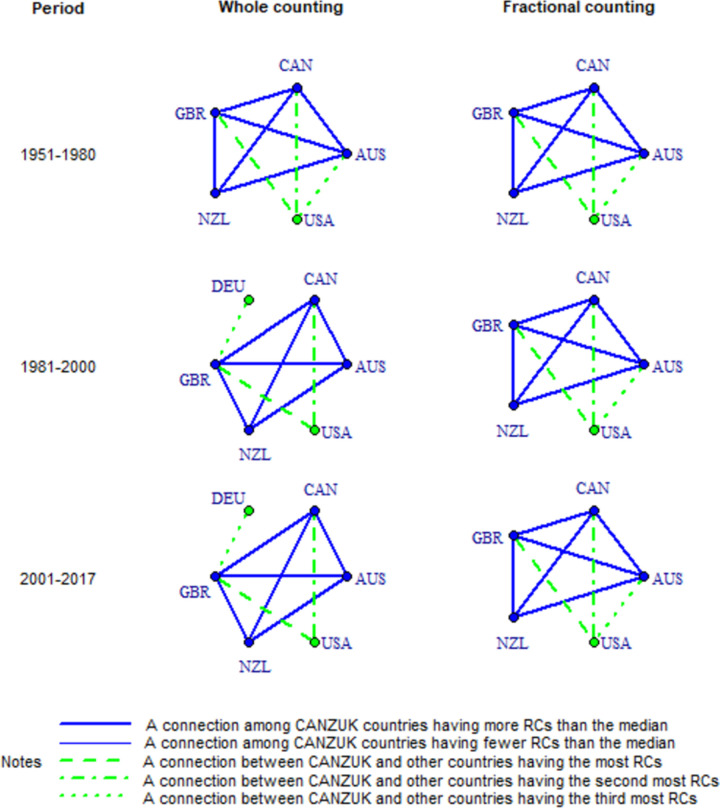
The co-authorship map of RC among CANZUK countries, and the top relations having the top three most active RCs between them and other countries in three separate periods during the time 1951–2017, calculated by absolute strength measure.

Fourthly, research connections among CANZUK countries have more RCs than the median values of all CANZUK’s RCs, as shown in Fig 4 and Tables 5–7. The images in Fig 4 show the ‘absolute strength’ of RC relationships among CANZUK countries and the top three bilateral research relations (i.e., having most active RCs) between CANZUK countries and outside countries (described as top three from now on). The relationships among CANZUK countries are presented as solid lines in blue, while the relationships between CANZUK countries and other countries are presented by dashed lines in green. The thicknesses of the lines in Fig 4 represent the relative strength of research connections to their median values.

**Table 5:** The ratios of whole/fractional counts to the corresponding medians during 1951–1980. Two groups of RCs were examined: RCs among CANZUK countries and the top three RCs between individual members of the CANZUK group and other countries.

	RCs among CANZUK countries						Top three RCs between CANZUK members and other countries		
**Country 1**	**AUS**	**AUS**	**AUS**	**CAN**	**CAN**	**GBR**	**AUS**	**CAN**	**GBR**
**Country 2**	**CAN**	**GBR**	**NZL**	**GBR**	**NZL**	**NZL**	**USA**	**USA**	**USA**
whole-count-to-median ratio (with the median value at 4)	57.25	151.75	52.75	227.75	16.50	48.00	214.00	703.50	905.50
fractional-count-to-median ratio (with the median value at 1.83)	58.80	156.66	56.36	233.49	17.01	50.59	220.90	741.34	940.66

**Table 6:** The ratios of whole/fractional counts to the corresponding medians during 1981–2000. Two groups of RCs were examined: RCs among CANZUK countries and the top three RCs between individual members of the CANZUK group and other countries.

	RC among CANZUK countries						Top three RCs between CANZUK members and other countries			
**Country 1**	**AUS**	**AUS**	**AUS**	**CAN**	**CAN**	**GBR**	**AUS**	**CAN**	**GBR**	**GBR**
**Country 2**	**CAN**	**GBR**	**NZL**	**GBR**	**NZL**	**NZL**	**USA**	**USA**	**DEU**	**USA**
whole-count-to-median ratio (with the median value at 15.5)	151.61	393.61	121.35	391.35	44.13	95.16		1,883.29	760.90	2,054.90
fractional-count-to-median ratio (with the median value at 5.63)	172.37	470.17	150.79	433.39	51.00	115.28	836.03	2,355.78		2,456.80

**Table 7:** The ratios of whole/fractional counts to the corresponding medians during 2001–2017. Two groups of RCs were examined: RCs among CANZUK countries and the top three RCs between the CANZUK members and other countries.

	RC among CANZUK countries						Top three RCs between CANZUK members and other countries			
**Country 1**	**AUS**	**AUS**	**AUS**	**CAN**	**CAN**	**GBR**	**AUS**	**CAN**	**GBR**	**GBR**
**Country 2**	**CAN**	**GBR**	**NZL**	**GBR**	**NZL**	**NZL**	**USA**	**USA**	**DEU**	**USA**
whole-count-to-median ratio (with the median value at 86)	277.98	600.37	172.69	514.67	58.58	130.44		1,851.66	1,052.00	2,076.38
fractional-count-to-median ratio (with the median value at 21.72)	356.66	851.29	271.31	641.45	72.17	184.62	1,366.81	2,968.24		3,011.78

Tables 5–7 below show a comparison of RCs’ strengths among CANZUK countries, and the top three bilateral research relations between CANZUK and other countries across the three periods. Because the numbers of RCs have increased over time (Table 5), we did not normalise the absolute values of RCs’ strengths by year for comparison across the three periods. Instead, we calculated the ratio of each relationship’s strength to the median strength of all relationships involving CANZUK countries in each period. These ratios were then used for comparison, as presented in Tables 5–7. These tables show that the ratios have increased over the three periods 1951–1980, 1981–2000, and 2001–2017. In other words, the strengths of RC relationships among CANZUK countries have become relatively more active. This indicates that the CANZUK countries increased their collaborations at a much higher speed than the collaborations of the rest of the world.

Fifthly, GBR has remained the top collaborator within the CANZUK group. Meanwhile, the USA was in the top research collaborations with the CANZUK group throughout the whole period (1951–1980). Only DEU (in the period 1981–2017) was also one in the top three of CANZUK’s important partners. These countries are also ranked by their RC weights, as represented in Tables 8–10. These tables show the RC weights of CANZUK countries and countries involved in the top three RCs with CANZUK countries. The value for each CANZUK country is the sum of all weights of RCs between that country and other CANZUK countries while the value of a non-CANZUK country is the sum of all weights of RCs between that country and a CANZUK country.

**Table 8:** Countries’ RC weights during 1951–1980, calculated by whole/fractional counting. Two groups of RCs were examined: RCs among CANZUK countries and the top three RCs between the CANZUK members and other countries.

	CANZUK countries				Countries involved in the top three RCs with CANZUK countries
	AUS	CAN	GBR	NZL	USA
Weight calculated by whole counting method	1,047.00	1,206.00	1,710.00	469.00	7,494.00
Weight calculated by fractional counting method	498.34	567.06	808.02	227.26	3,584.57

**Table 9:** Countries’ RC weights during 1981–2000, calculated by whole/fractional counting. Two groups of RCs were examined: RCs among CANZUK countries and the top three RCs between the CANZUK members and other countries.

	CANZUK countries				Countries involved in the top three RCs with CANZUK countries	
	AUS	CAN	GBR	NZL	DEU	USA
Weight calculated by whole counting method	10,332.00	9,100.00	13,642.00	4,040.00	18,370.00	74,212.00
Weight calculated by fractional counting method	4,466.27	3,697.41	5,735.81	1,785.06		32,878.97

**Table 10:** Countries’ RC weights during 2001–2017, calculated by whole/fractional counting. Two groups of RCs were examined: RCs among CANZUK countries and the top three RCs between the CANZUK members and other countries.

	CANZUK countries				Countries involved in the top three RCs with CANZUK countries	
	AUS	CAN	GBR	NZL	DEU	USA
Weight calculated by whole counting method	90,389.00	73,206.00	107,112.00	31,107.00	154,553.00	434,746.00
Weight calculated by fractional counting method	32,135.99	23,251.28	36,439.47			165,497.81

Lastly, the two methods (whole counting and fractional counting) give dissimilar RC strength rankings. For example, the whole counting method shows that the USA and DEU are the two top partners of the CANZUK group in the period 1981–2017 whereas the fractional counting method presents the USA as the only top partner during this period. The difference occurs because DEU had more collaborations with CANZUK countries and therefore was credited with more whole counts. Meanwhile, the USA was credited with more collaboration proportions because their co-authored publications counted fewer total partners.

### Bilateral similarity of CANZUK’s RCs

From analysing the absolute strengths of CANZUK’s RCs as in the above section, we can observe that the numbers of co-authored publications have increased over time. However, this apparent growth may not be due to any change in the nature of collaborations between these countries but simply in general growth in these countries. For example, several factors could explain this, including population growth and the increase in the number of scientists in these countries, among others. Therefore, we applied a normalisation of these numbers. The bilateral similarity approach normalises these numbers using the total number of IRC papers published by the two countries in a collaboration pair.

**Fig 5:**
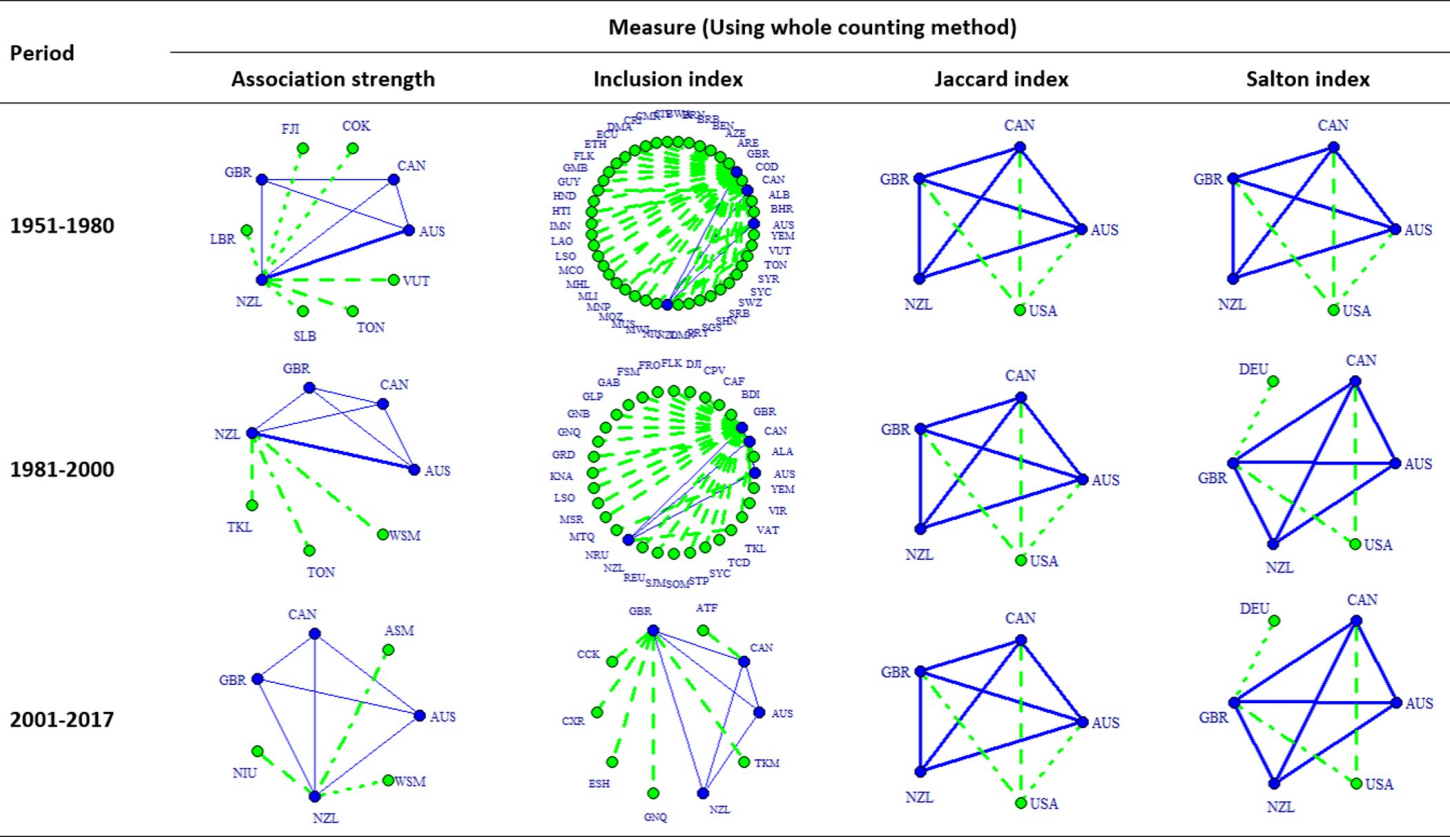
The co-authorship map of RC among CANZUK countries, and the top relations having the top three most active RCs between them and other countries in three separate periods during the time 1951–2017, calculated by four bilateral similarity measures, using the whole counting method.

**Fig 6:**
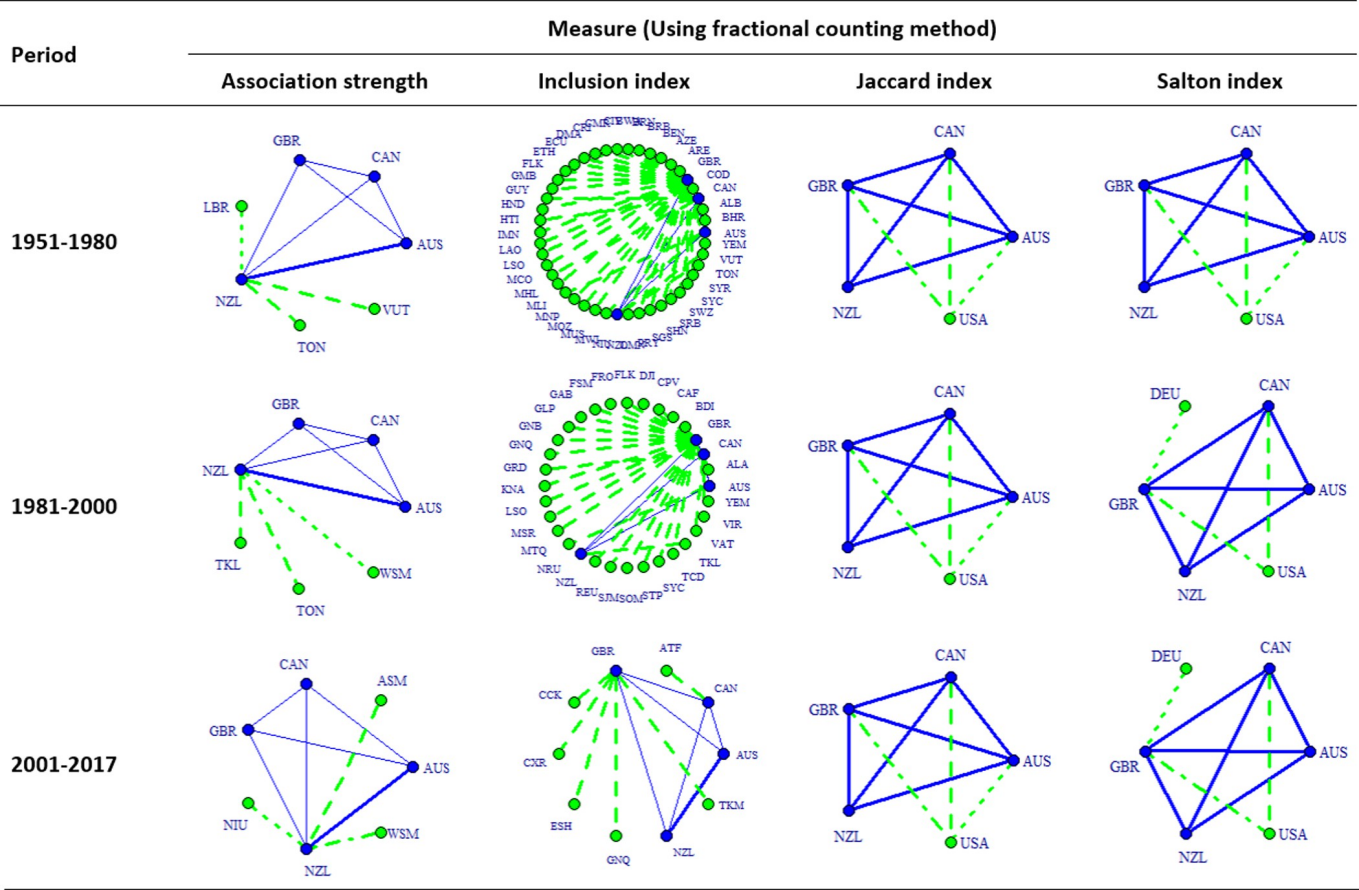
The co-authorship map of RC among CANZUK countries, and the top relations having the top three most active RCs between them and other countries in three separate periods during the time 1951–2017, calculated by four bilateral similarity measures, using the fractional counting method.

Figs 5 and 6 present the co-authorship maps of RC among CANZUK countries, calculated using the whole counting method and the fractional counting method, respectively. Each of these figures visualises the comparison between the results calculated by four applied measures: association strength, inclusion index, Jaccard index, and Salton index. Examining each figure, the patterns of co-authorship maps produced by the Jaccard index and by the Salton index are quite similar, yet distinct from the patterns of co-authorship maps generated by the association strength and inclusion index.

Upon comparing Figs 5 and 6, it becomes evident that the resulting networks portrayed in these two figures are similar when generated using the same measures, except for the ‘association strength’ measure. The utilisation of the association strength measure, combined with either the whole counting method or the fractional counting method, generates slightly different patterns in Figs 4 and 5. The image produced using the whole counting method in this case shows that the five most important collaborations with outside countries are those between the NZL and COK, FJI, LBR, SLB, and TON. On the other hand, the image produced by implementing the fractional counting method shows only three top relations, namely between NZL and LBR, TON, and VUT. Additionally, the image produced using the fractional counting method highlights that AUS and NZL exhibit a stronger collaboration compared to other collaborations among CANZUK countries during the period 2001–2017. However, the image produced by implementing the whole counting method does not demonstrate this phenomenon.

It is also worth noting that there are some similarities in the images generated by using measures to evaluate the absolute strengths, bilateral strengths, and multilateral strengths of CANZUK’s RCs. Specifically, the association strength measure results in images that are similar to those from the multilateral similarity measure (as presented in the following section). Meanwhile, the Jaccard index and Salton index produce images similar to those from the ‘absolute strength’ measure. For example, one notable finding is that the USA has consistently remained a top partner in the RCs with two countries in the CANZUK group ‐ CAN and GBR. These findings can be observed when comparing the images resulting from using the ‘absolute strength’ measure (Fig 4), and those resulting from using the Jaccard index and Salton index (Figs 5 and 6).

However, there are some differences between the results obtained from either the Jaccard index, the Salton index, or the ‘absolute strength’ measure. The most notable finding from the two former measures is that CAN was the CANZUK country that the USA had the strongest research relationship with. This observation is different from the findings obtained from the evaluation of the CANZUK’s RCs’ absolute strengths, which show that the USA had the strongest research relationship with GBR.

The two measures calculating the bilateral similarity of CANZUK’s RC, the Salton index and the Jaccard index, also reflect some slightly different details in the collaboration maps. For the Salton index, the USA and DEU were the two countries that had the three strongest research relationships with CANZUK countries in the period 2001–2017. By contrast, the Jaccard index shows the USA was the only country that had the three strongest research relationships with CANZUK countries. The Jaccard index, therefore, reflects similar results to those obtained from the absolute strengths of CANZUK’s RC with fractional counting, while the Salton index shows similar results with the absolute strengths using the whole counting.

Table 11 shows the values of the Jaccard index and the Salton index of CANZUK’s RCs. In this table, the higher the values are, the more active the RCs are when the sizes of IRC publications of the two relevant countries are applied to normalise these strength values.

**Table 11:** The bilateral similarity of CANZUK’s RCs in three separate periods between 1951–2017. The values show that bilateral similarity has decreased over time, except for the case of the AUS-CAN research collaboration from the period 1981–2000 to the period 20012017 (calculated by the Jaccard index), and the GBR-NZL research collaboration from the period 1981–2000 to the period 2001–2017 (calculated by both the Salton index and the Jaccard index).

Country 1			AUS	AUS	AUS	CAN	GBR	GBR
Country 2			CAN	GBR	NZL	NZL	CAN	NZL
Calculated by Salton index	Using whole counting method	1951–1980	0.057	0.110	0.139	0.029	0.110	0.062
		1981–2000	0.042	0.078	0.092	0.025	0.057	0.038
		2001–2017	0.045	0.070	0.065	0.012	0.054	0.032
	Using fractional counting method	1951–1980	0.058	0.113	0.147	0.029	0.112	0.065
		1981–2000	0.042	0.083	0.100	0.025	0.056	0.041
		2001–2017	0.044	0.078	0.084	0.012	0.052	0.038
Calculated by Jaccard index	Using whole counting method	1951–1980	0.027	0.046	0.063	0.010	0.056	0.016
		1981–2000	0.020	0.033	0.038	0.008	0.028	0.009
		2001–2017	0.023	0.033	0.026	0.07	0.026	0.009
	Using fractional counting method	1951–1980	0.027	0.047	0.068	0.010	0.057	0.017
		1981–2000	0.020	0.036	0.042	0.008	0.028	0.010
		2001–2017	0.022	0.037	0.032	0.007	0.026	0.010

### Multilateral similarity of CANZUK’s RCs

**Fig 7:**
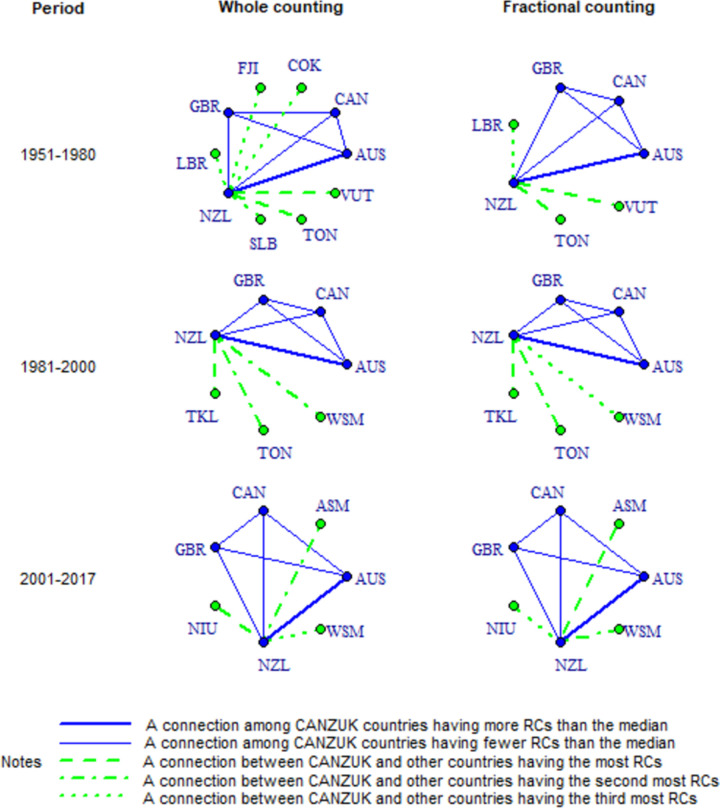
The co-authorship map of RC among CANZUK countries, and the top relations having the top three most active RCs between them and other countries in three separate periods during the time 1951–2017, calculated by multilateral similarity measure.

Table 12 shows the comparison of CANZUK’s RCs in the three periods, calculated by taking all the countries’ research sizes to normalise the RCs’ strength values. Those values greater than 1 mean that the corresponding research connections have more RCs than expected. Those values less than 1 mean that the corresponding research connections have fewer RCs than expected. Fig 7 shows the collaboration maps in these periods. This figure shows that the RCs between NZL and some other countries are the mainstream pattern.

**Table 12:** The multilateral similarity of CANZUK’s RCs in three separate periods during `the time 1951–2017. The values show that multilateral similarity has decreased over time.

Country 1		AUS	AUS	AUS	CAN	GBR	GBR
Country 2		CAN	GBR	NZL	NZL	CAN	NZL
Using whole counting method	1951–1980	0.53	0.75	3.41	0.47	0.50	0.74
	1981–2000	0.38	0.51	2.30	0.46	0.28	0.51
	2001–2017	0.36	0.41	1.24	0.34	0.29	0.40
Using fractional counting method	1951–1980	0.53	0.77	3.61	0.48	0.50	0.78
	1981–2000	0.38	0.54	2.47	0.45	0.27	0.54
	2001–2017	0.35	0.45	1.65	0.35	0.27	0.49

Fig 7 reveals noticeable distinctions between the collaboration maps derived from CANZUK’s RCs using the multilateral similarity measure, as compared to both the collaboration maps based on the absolute strength measure and those based on the bilateral similarity measure of CANZUK’s RCs. In this figure, the thick lines describe connections among CANZUK countries having more observed RCs than expected while the thin lines describe connections among CANZUK countries having fewer observed RCs than expected. In terms of expectation, only the relationship between AUS and NZL has had more RCs than expected. Fig 7 also shows that the top collaborators with CANZUK countries, in terms of expectation, have changed dramatically over time. In the period 1951–1980, NZL had top research connections with the Cook Islands, Fiji, Liberia, Tonga, Vanuatu, and the Solomon Islands; or just Liberia, Tonga, and Vanuatu, depending on whether the whole counting method or the fractional counting method was applied in calculating multilateral similarities. In the period 1981–2000, Samoa, Tokelau, and Tonga were the top collaborators with the CANZUK countries through RCs with NZL. In the period 2001–2017, NZL had top research connections with Samoa, American Samoa, and Niue. We also carried out chi-square goodness-of-fit tests to compare the observed distribution to the expected distribution of RCs. Table 13 shows that only the chi-square tests’ p-value of the test using fractional counting for the period 2001–2017 is higher than 0.05, which means that generally there is a significant difference between the observed and the expected RCs at the 5% significance level. In other words, the thicknesses of the lines in Fig 7 are meaningful because their corresponding ratios (of observed values to expected values) are significantly different from 1.

**Table 13:** The results of chi-square goodness of fit tests comparing the observed distribution to an expected distribution.

Period	Using whole counting method	Using fractional counting method
1951–1980	X-squared = 26646, df = 19588 p-value < 2.2e-16	X-squared = 67305, df = 58520, p-value < 2.2e-16
1981–2000	X-squared = 140827, df = 116052, p-value < 2.2e-16	X-squared = 327377, df = 315758, p-value < 2.2e-16
2001–2017	X-squared = 327377, df = 315758, p-value < 2.2e-16	X-squared = 768873, df = 768012, p-value = 0.2435

In summary, the various measurement approaches for IRC produce different findings. Table 14 provides an overview of the measures employed and their corresponding results.

**Table 14:** The specific measures employed for each of the three approaches: Absolute strength, bilateral similarity, and multilateral similarity, along with the corresponding results obtained.

Approach	Specific measure	Result
Absolute strength	Observed frequencies of RCs	Research collaborations among CANZUK countries have increased over time, as evidenced by rising collaboration pairs and co-authored publications, indicating a strengthening of ties within the group. Additionally, collaborations between CANZUK countries and other nations demonstrate a pattern of outlier-prone relationships, suggesting a significant presence of outlier collaborations outside the CANZUK group.
Bilateral similarity	Association strength	The co-authorship maps generated using different measures and counting methods reveal consistent patterns among CANZUK countries. The association strength measure results in images that are similar to those from the multilateral similarity measure, while the Jaccard index and Salton index produce images similar to those from the absolute strength measure. Collaborations between the CANZUK group and other countries reveal variations in collaboration patterns and top partnerships, indicating differences in research relationships and partnership strengths across different measures.
	Inclusion index	
	Jaccard index	
	Salton index	
Multilateral similarity	Ratios of observed frequencies to expected frequencies of RCs	Research collaborations among CANZUK countries reveal varying levels of collaboration strength, with some connections having more observed collaborations than expected while others have fewer, indicating fluctuations in research ties within the group over time. Meanwhile, collaborations between the CANZUK group and other countries exhibit changing patterns of top collaborators, highlighting shifts in research partnerships and significant disparities between observed and expected collaborations.

## Discussion

The goal of our investigation is to examine the research collaborations of CANZUK countries. Here we discuss the findings of this study. The discussion is structured around the research questions and related to previous research. Additionally, this section ends with a summary detailing how the results support the paper’s objectives.

### RQ1: How have the RCs among CANZUK countries developed over time?

The RCs among CANZUK countries have been a symmetrical network, meaning it is evolving towards complete connectedness and equal distribution. Since this network symmetry relates to system robustness [35], we infer that the RCs among CANZUK countries have been in a more sustainable network, in which every country has diversified and more balanced relationships with other countries in the group. A sustainable network state is a preferred state for “physical (constructed and natural) systems and human communities (social and institutional)” in order to “possess the capacity to survive, cope, recover, learn and transform from disturbances’’ [36], or to "be able to survive and function under extreme stress" [37]. However, this development is still below what is possible because the numbers of IRC publications between the CANZUK countries are relatively low in comparison with each country’s total IRC publications. The only exceptional case, in terms of expectation, is the strong collaboration between AUS and NZL. This strong collaboration is consistent with another study’s findings for the period 1999–2005 [4].

### RQ2: How have the RCs between CANZUK and other countries developed over time?

The RCs between CANZUK and other countries have also developed during the years under investigation, but the top collaborating partners have been mainly determined by the top collaborators [32] within the CANZUK group. The more active RCs the UK and Canada, and the USA reflect the traditionally close economic and political links between these nations. In general, the strong research partnerships between the CANZUK group and the USA have been explained by their historical relationships [7, 16], and as a result of “The Technical Cooperation Programme" comprising of the USA, UK, Canada, Australia and New Zealand [3, 38].

By contrast, the other positions of the top partners in each period reflect the RC policy in CANZUK countries during these times. Depending on the measure used, DEU might be considered having one of the three top RCs with the CANZUK. Since the former East German states joined the Federal Republic of Germany in 1990, Germany gradually strengthened its research capacity and became an important research partner (after the USA) with CANZUK countries, using the whole counting method by Salton index. Although there has been an increase in research collaborations between China and the most developed countries in the world, including England and Canada [24], as a result of China’s reform policy, China was not in the three top research partners with CANZUK. Previous studies have separately mentioned the strong research relationships between some of the CANZUK countries and Germany, and the USA [8, 16]. For example, the study by Zitt et al. [16] describes the research collaboration among five countries having large scientific research sectors, namely France, Germany, Japan, the United Kingdom, and the United States. This study includes the descriptions about the research collaborations between one of the CANZUK countries, the United Kingdom, with other countries, using the 3-year-average values of the two separate periods, 1996 (1995–1997) and 1986 (1985–1987). By contrast, our study examines and presents the RCs between all CANZUK countries and other countries throughout the period 1951–2017, which covers the time coverage in a previous study [32]. In other words, our study gives an overall picture of the development of RCs between CANZUK and other countries. Therefore, our study provides beneficial values for researchers and policy makers who want to have a systematic overview of the IRCs between the CANZUK group and other countries. A complete picture of RCs between countries is essential for identifying strategic collaborations [39] so that countries can raise their innovation competitiveness [40]. Our study also finds that the actual collaborating outcomes may be interpreted differently, depending on the approach applied. For example, the multilateral similarity measure approach considers the research relations with all countries’ RCs. In this approach, different periods see different CANZUK countries performing RC with others over what is expected. These differences were described in the next research question.

### RQ3: To what extent do the different measures of RC reveal different IRCs?

The measures applied in this study show different patterns of RC development and different patterns of RC rankings. Figs 4–7 show different results obtained by different approaches to measuring the RCs’ strengths: by absolute strength measure, by bilateral similarity measures and by multilateral similarity measure. On the one hand, the absolute strength measure shows that research relationships among CANZUK countries have developed over time. In detail, this measure shows that the research relationships among CANZUK countries have had more RCs in average (compared to the median value) and these research relationships have also increased their relatively normalised strengths. On the other hand, the bilateral similarity measures and the multilateral similarity measure show that the relative strengths of research relationships among CANZUK countries have developed less over time.

However, the ranks of countries having RCs with the CANZUK countries by these measures are similar: the USA has consistently been the most popular country for CANZUK countries to partner with, and GBR is the most collaborating country in the CANZUK group.

It is worth noting that there are differences in patterns of RC development and rankings given by these above measures, and these differences can be explained by the nature of different ways of calculation. For the absolute strength measure, the Jaccard index, and the Salton index: the numbers of RCs between each pair of countries are not normalised or are normalised by the ‘average’ size of the two corresponding countries’ IRC publications. For the second group (the association strength measure and the multilateral similarity measure): the numbers of RCs between each pair of countries are normalised using the product of the two corresponding countries’ amounts of IRC publications. Therefore, the association strength measure and the multilateral similarity measure tend to give higher scores for the research relationships between two countries having more gaps in the amounts of IRC publications (e.g., one country has a low number of IRC publications while the other country has a high number of IRC publications), which can be seen in the cases of NZL in comparison with its research partners (Figs 5 and 7).

Previous research has suggested that the impacts of different counting methods should be considered in IRC measurement studies. For each measure in this study, the two underlying counting methods applied also lead to slight differences in particular rankings. An example is the case of the absolute RC strength measure presented in Fig 4. In this figure, the ranking of the top collaborating countries with the CANZUK group varies for each of the examined periods, depending on whether the whole counting method or fractional counting method is used. The three top strongest RCs feature different CANZUK partners (the USA only, or the USA and DEU).

The differences in rankings given by the whole counting method and the fractional counting method can also be explained by the nature of their calculation. The former is more suitable for studies focusing on the number of research relationships between co-authors, while the latter is more suitable for studies focusing on the number of publications credited to the countries involved in the research collaboration. These differences in the results obtained by applying these two methods are notable because they tell different stories about the CANZUK RCs. For example, the whole counting method shows that the CANZUK group still has more active RCs with their traditional European partner, DEU, while the fractional counting method shows that the USA has surpassed DEU to become the only top research partner of CANZUK countries in the period 1981–2017.

The substantial differences observed in the rankings of the top collaborators with CANZUK countries are consistent with findings in previous literature. For example, research studying the problem of multiple authorship [41] shows that the difference between whole counting and fractional counting is small when measuring low frequency collaborators, but that the difference becomes substantial when measuring high frequency collaborators. The difference is caused because the whole counting method amplifies the credits of countries having more RCs in multi-authored publications. The fractional counting reduces this bias by dividing each publication by the number of co-authors [41]. As both methods have biases [29], researchers prefer to use either the whole counting [12] or the fractional counting method [16, 42]. This study measures the collaborative strength of CANZUK countries so both underlying counting methods are useful to examine two different aspects of CANZUK’s collaborative strengths: the former describes the relationship-based strengths while the latter describes the production-based strengths.

The other findings suggest two recommendations for choosing suitable measures of research collaboration strengths. First, the choice of measures should depend on the purpose of measurement. The absolute strength is suitable for comparing the numbers of RCs between countries. The bilateral similarity measures are suitable for comparing the relative strengths of research relationships between pairs of countries, in relation to the two corresponding countries’ sizes of IRC publications. The multilateral similarity measure is preferable when examining whether the ‘observed’ strengths of research relationships between pairs of countries meet their ‘expected’ values. Second, the Jaccard index and the Salton index can be considered for alternative use if the purpose of IRC measurement is to compare the relative strengths of research relationships. For example, a study applying the Salton index measure about international research collaboration in the period 1981–1985 [13] showed that the USA had strong collaboration links with the UK and Canada. This study would have had significantly different results if the authors had used a different measure, such as association strength or inclusion index (as illustrated in Figs 5 and 6).

In summary, we aimed to analyse the evolution of CANZUK research collaborations over time and assess their strength using various measures. To achieve this, we examined statistical methods related to research collaborations and employed three distinct measurement approaches: ’absolute strength’, ’bilateral similarity’, and ’multilateral similarity’. Our analysis of descriptive statistics revealed that CANZUK countries have developed notably, both in the total number of collaboration pairs and in the median values of co-authored publications among CANZUK countries when compared to other nations, forming a more balanced research network. However, the growth of these research collaborations was not as much as expected. Differences between the observed and expected values of CANZUK’s research collaborations were supported by chi-square goodness-of-fit tests. The results suggest a growing trend in research collaboration relationships among CANZUK countries over time, with the potential for further development.

Furthermore, the three measurement approaches consistently identified the USA as the top outside collaborator of the CANZUK countries. Pivotal collaborators within and beyond the CANZUK group can be explained by historical, economic, and political influences.

Moreover, the application of the three measurement approaches to assess collaboration strengths revealed diverse patterns and rankings of countries involved in research collaborations. Despite variations, notable similarities emerged in the results. Specifically, the association strength measure of ’bilateral similarity’ produced outcomes similar to those obtained through the ’multilateral similarity’ measure. Conversely, the Jaccard index and Salton index of ’bilateral similarity’ exhibited similarities with the ’absolute strength’ measure.

## Conclusion and future work

In conclusion, the present study aimed to find out how the RCs of CANZUK countries have developed over time. Our findings suggest that these collaborations get better over time, demonstrating a balanced growth in bilateral partnerships within the CANZUK group, as well as an increase in active collaborations with other nations. In addition, different measures gave notably different results when they were used to examine the strengths of RCs between countries. The CANZUK’s collaboration map and the ranking lists of top collaborating countries with the CANZUK group were depicted differently depending upon the measures used. These differences might create conflicting conclusions in RC studies. Therefore, the choice of suitable measures should depend on the purpose of the RC studies.

There are limitations in the present study and opportunities to explore further. Firstly, this study chose the MAG data source to examine the research collaborations of CANZUK countries and the results from this choice may be different than those obtained if other data sources were used. A study about the effects of data set choice on measuring IRC has shown that different data sources give slightly different outcomes of IRC measurement [43]. Therefore, finding which data source is the most suited for IRC measurement and its corresponding statistics of CANZUK’s research collaborations should be another focus of IRC studies. Secondly, this study used co-authored publications as the common outcomes of research collaborations. However, there are various types of outcomes beyond joint research publications: patents, joint research grants, etc. [44], and different rewards [45] of the collaborations: acknowledgements in PhD theses, research journals (articles, editorials, reviews, etc.) and books. The use of only co-authored publications could have overlooked the cases where there was no co-authored publication; or reflected just a part of the research collaboration between countries. These other potential outcomes, together with co-authored publications, should be examined in future work to reveal a more accurate picture of the CANZUK RC network.

Despite the limitations, the present study is important because it provides insights into the RCs between CANZUK countries. We are explorers in studying the research collaboration of the CANZUK countries as a group and our exploration has shown that their shared histories of language, cultural tradition, and economic developments can encourage their research connections although they do not have close geographical proximity. Therefore, the depth and quality of these partnerships undoubtedly improve with time, much like the refinement of wine with age. Our study is also a pioneering work in applying and comparing different measures of IRC assessment. For research policy, the study shows that the RCs among CANZUK countries have been in a balanced network but these RCs perhaps remain below what is possible, in relation to their numbers of IRC publications. In other words, the CANZUK countries could have further strengthened their research collaborations. These strong research collaborations are increasingly important for the CANZUK countries, especially as the UK has left the European Union (i.e., needs to review its research partnerships) and Australia is looking to strengthen its collaborations with long-established allies via agreements such as the trilateral security pact (AUKUS) with the United Kingdom and the United States [46]. The circumstances mentioned above can encourage policy makers to support further research collaborations among CANZUK countries.

## Supporting information

S1 Table Explanation for the list of the current officially assigned ISO 3166–1 alpha-3 codes and their corresponding English country (or dependent territory) names in the present study. (DOCX)
