# Supplementary material for: Do research collaborations age like wine? Absolute and relative measures of CANZUK research partnerships’ strength since the 1950s
Source: PLoS One. 2024 Apr 16;19(4):e0299319. doi: 10.1371/journal.pone.0299319 (PMC11020478; doi:10.1371/journal.pone.0299319)
Supplement: S1 Table — (DOCX) [file pone.0299319.s001.docx]

**S1 Table**

| **ISO 3166-1 alpha-3 codes** | **English country (/dependent territory) names** |
| --- | --- |
| ARM | Armenia, officially the Republic of Armenia |
| AUS | Australia, officially the Commonwealth of Australia |
| BGR | Bulgaria, officially the Republic of Bulgaria |
| BHR | Bahrain, officially the Kingdom of Bahrain |
| BHS | The Bahamas, known officially as the Commonwealth of The Bahamas |
| BIH | Bosnia and Herzegovina |
| BLR | Belarus, officially the Republic of Belarus |
| BRB | Barbados |
| BRN | Brunei, officially the Nation of Brunei, the Abode of Peace |
| BTN | Bhutan, officially known as the Kingdom of Bhutan |
| BWA | Botswana, officially the Republic of Botswana |
| CAN | Canada |
| CHE | Switzerland, officially the Swiss Confederation |
| CHN | China, officially the People's Republic of China |
| CMR | Cameroon, officially the Republic of Cameroon |
| DEU | Germany, officially the Federal Republic of Germany |
| EGY | Egypt, officially the Arab Republic of Egypt |
| ETH | Ethiopia, officially the Federal Democratic Republic of Ethiopia |
| FJI | Fiji, officially the Republic of Fiji |
| FRA | France, officially the French Republic |
| GBR | The United Kingdom of Great Britain and Northern Ireland, commonly known as the United Kingdom (UK) or Britain |
| GGY | The Bailiwick of Guernsey |
| GHA | Ghana, officially the Republic of Ghana |
| GRC | Greece, officially the Hellenic Republic |
| GTM | Guatemala, officially the Republic of Guatemala |
| GUM | Guam |
| HRV | Croatia, officially the Republic of Croatia |
| HUN | Hungary |
| IDN | Indonesia, officially the Republic of Indonesia |
| IRL | Ireland, officially known as the Republic of Ireland |
| IRN | Iran, officially the Islamic Republic of Iran |
| IRQ | Iraq, officially the Republic of Iraq |
| ISR | Israel, officially known as the State of Israel |
| ITA | Italy, officially the Italian Republic |
| JOR | Jordan, officially the Hashemite Kingdom of Jordan |
| KAZ | Kazakhstan, officially the Republic of Kazakhstan |
| KWT | Kuwait, officially the State of Kuwait |
| LCA | Saint Lucia |
| LIE | Liechtenstein, officially the Principality of Liechtenstein |
| LKA | Sri Lanka, officially the Democratic Socialist Republic of Sri Lanka |
| LUX | Luxembourg, officially the Grand Duchy of Luxembourg |
| MAR | Morocco, officially the Kingdom of Morocco |
| MCO | Monaco, officially the Principality of Monaco |
| MDA | Moldova, officially the Republic of Moldova |
| MEX | Mexico, officially the United Mexican States |
| MLT | Malta, officially known as the Republic of Malta |
| MNE | Montenegro |
| MUS | Mauritius, officially the Republic of Mauritius |
| MWI | Malawi, officially the Republic of Malawi |
| MYS | Malaysia |
| NER | Niger, officially the Republic of the Niger |
| NFK | Norfolk Island |
| NGA | Nigeria, officially the Federal Republic of Nigeria |
| NLD | The Netherlands |
| NOR | Norway, officially the Kingdom of Norway |
| NPL | Nepal, officially the Federal Democratic Republic of Nepal |
| NZL | New Zealand |
| OMN | Oman, officially the Sultanate of Oman |
| PAK | Pakistan, officially the Islamic Republic of Pakistan |
| PAN | Panama, officially the Republic of Panama |
| PER | Peru, officially the Republic of Peru |
| POL | Poland, officially the Republic of Poland |
| PRK | North Korea, officially the Democratic People's Republic of Korea (DPRK) |
| PRT | Portugal, officially the Portuguese Republic |
| PRY | Paraguay, officially the Republic of Paraguay |
| PSE | Palestine, officially the State of Palestine |
| SAU | Saudi Arabia, officially the Kingdom of Saudi Arabia (KSA) |
| SDN | Sudan, officially the Republic of the Sudan |
| SEN | Senegal, officially the Republic of Senegal |
| SRB | Serbia, officially the Republic of Serbia |
| SVN | Slovenia, officially the Republic of Slovenia |
| THA | Thailand |
| TTO | Trinidad and Tobago, officially the Republic of Trinidad and Tobago |
| URY | Uruguay, officially the Oriental Republic of Uruguay |
| USA | The United States (U.S. or US) or America, officially the United States of America (U.S.A. or USA) |
| VCT | Saint Vincent and the Grenadines |
| VEN | Venezuela, officially the Bolivarian Republic of Venezuela |
| WSM | Samoa, officially known as the Independent State of Samoa |
| YEM | Yemen, officially the Republic of Yemen |
